# Deciphering the miRNA transcriptome of breast muscle from the embryonic to post-hatching periods in chickens

**DOI:** 10.1186/s12864-021-07374-y

**Published:** 2021-01-19

**Authors:** Jie Liu, Fuwei Li, Xin Hu, Dingguo Cao, Wei Liu, Haixia Han, Yan Zhou, Qiuxia Lei

**Affiliations:** 1 Shandong Academy of Agricultural Sciences, Poultry Institute, Ji’nan, 250023 China; 2 Poultry Breeding Engineering Technology Center of Shandong Province, Ji’nan, 250023 China; 3 Shandong Provincial Key Laboratory of Poultry Diseases Diagnosis and Immunology, Ji’nan, 250023 China; 4 Key Laboratory of Animal (Poultry) Genetics Breeding and Reproduction, Ministry of Agriculture and Rural Affairs, Beijing, 100193 China; 5 Molecular and Cellular Biology, Gembloux Agro-Bio Tech, University of Liège, Gembloux, 5030 Belgium

**Keywords:** Breast muscle, Muscle development, miRNA transcriptome, Differential expression profiles

## Abstract

**Background:**

miRNAs play critical roles in growth and development. Various studies of chicken muscle development have focused on identifying miRNAs that are important for embryo or adult muscle development. However, little is known about the role of miRNAs in the whole muscle development process from embryonic to post-hatching periods. Here, we present a comprehensive investigation of miRNA transcriptomes at 12-day embryo (E12), E17, and day 1 (D1), D14, D56 and D98 post-hatching stages.

**Results:**

We identified 337 differentially expressed miRNAs (DE-miRNAs) during muscle development. A Short Time-Series Expression Miner analysis identified two significantly different expression profiles. Profile 4 with downregulated pattern contained 106 DE-miRNAs, while profile 21 with upregulated pattern contained 44 DE-miRNAs. The DE-miRNAs with the upregulated pattern mainly played regulatory roles in cellular turnover, such as pyrimidine metabolism, DNA replication, and cell cycle, whereas DE-miRNAs with the downregulated pattern directly or indirectly contributed to protein turnover metabolism such as glycolysis/gluconeogenesis, pyruvate metabolism and biosynthesis of amino acids.

**Conclusions:**

The main functional miRNAs during chicken muscle development differ between embryonic and post-hatching stages. miRNAs with an upregulated pattern were mainly involved in cellular turnover, while miRNAs with a downregulated pattern mainly played a regulatory role in protein turnover metabolism. These findings enrich information about the regulatory mechanisms involved in muscle development at the miRNA expression level, and provide several candidates for future studies concerning miRNA-target function in regulation of chicken muscle development.

**Supplementary Information:**

The online version contains supplementary material available at 10.1186/s12864-021-07374-y.

## Background

Chicken skeletal muscle constitutes the largest proportion and most valuable component of meat mass; its development is closely associated with the amount of meat production and its quality. Skeletal muscle development is a complex multi-process trait regulated by various genetic factors, including gene polymorphism, transcription factors, DNA methylation and noncoding RNAs (ncRNAs) [1–4]. These genetic factors co-operate with each other to ensure normal development of skeletal muscle.

miRNAs, an important type of ncRNAs, are proposed to control or fine-tune complex genetic pathways by post-transcriptional regulation of target genes [5, 6]. miRNAs have been found to have important regulatory roles during skeletal muscle development [3]. For example, miR-1, miR-133 and miR-206 are specifically and abundantly expressed in muscle tissue and contribute to muscle development. miR-1 and miR-133 are involved in myoblast proliferation and differentiation [7], and miR-206 has been shown to promote myoblast differentiation [8, 9]. Skeletal muscle development is a multi-step process that includes myofiber formation and hypertrophy. Cellular turnover plays a major role in the formation of myofiber, which occurs mainly in embryogenesis [10, 11]. After myofibers are formed, they undergo hypertrophy at the postnatal stage [12]. Postnatal muscle hypertrophy is mainly associated with accumulation of muscle-specific proteins [13]. In addition to these complex cell developmental processes during myofiber formation and hypertrophy, the fine-tuned regulation of numerous myogenic genes is also important for the development of skeletal muscle [4]. Our previous study also showed that there were distinct gene regulatory mechanisms of chicken muscle development between the embryonic and post-hatching periods, based on RNA sequencing of breast muscle tissue obtained from Shouguang chickens at 12-day embryo (E12), E17 and day 1 (D1), D14, D56 and D98 post-hatching stages [14]. However, a comprehensive study of the dynamics of miRNAs during chicken muscle development is lacking, especially from embryonic to post-hatching period. Most of the previous studies have focused on the dynamics of miRNAs in the embryonic or post-hatching period. For example, Jebessa et al. explored the miRNA expression profile during chicken leg muscle development at E11, E16 and D1 [15], while Li et al. analyzed miRNA and mRNA expression profiles during chicken breast muscle development at 6, 14, 22 and 30 weeks of age [16].

To elucidate systematically the molecular mechanisms underlying chicken muscle development, we performed miRNA sequencing to explore the miRNA profile in breast muscle of Shouguang chickens from the embryonic to post-hatching periods (E12, E17, D1, D14, D56 and D98), which will help us to explore the development-related miRNA expression signatures in breast muscle and improve our understanding of the regulatory mechanism of miRNAs in muscle development.

## Results

### Analysis of small RNAs

**Fig. 1:**
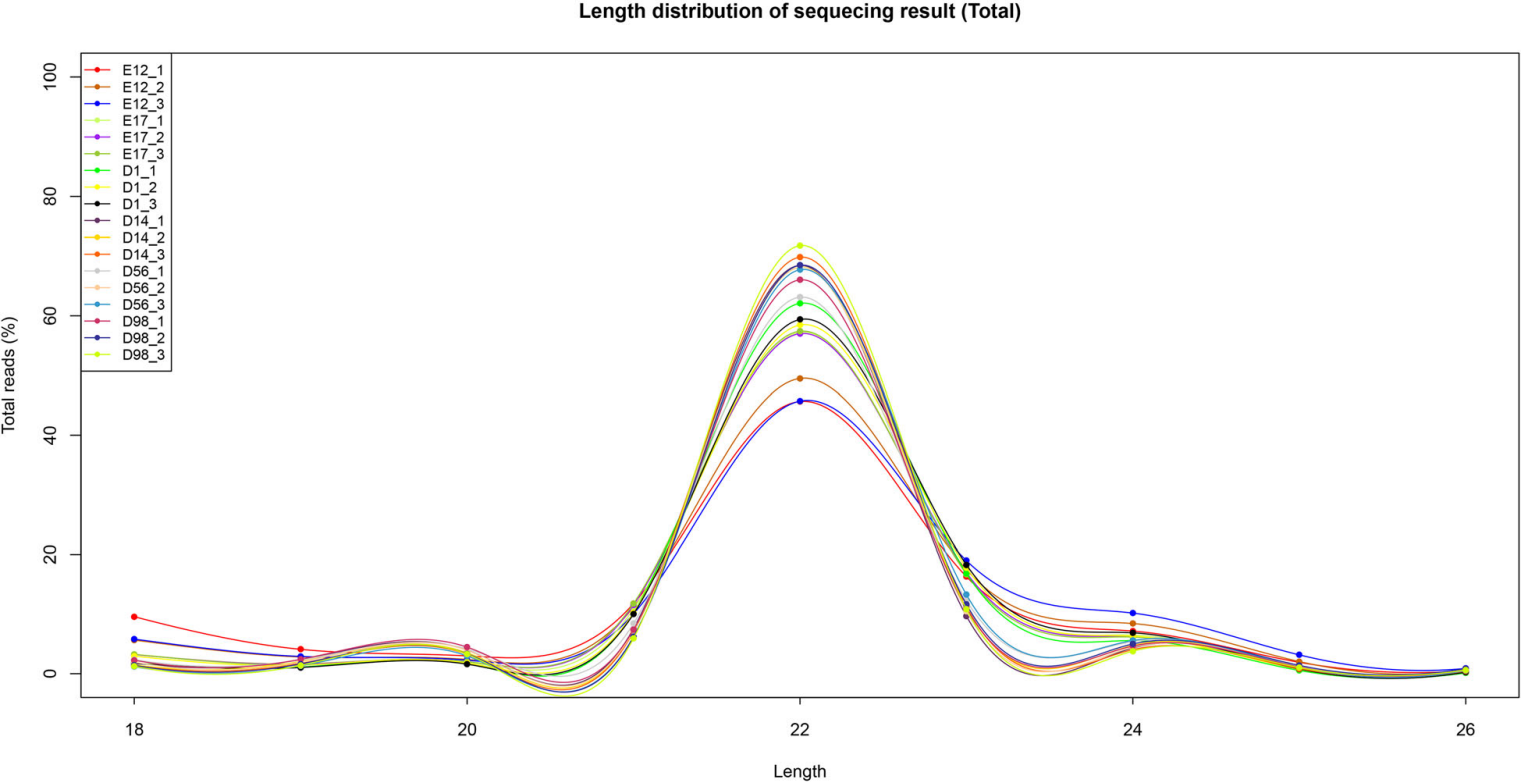
Length distribution of sequenced small RNA reads

We established 18 small RNA libraries (E12_1, E12_2, E12_3, E17_1, E17_2, E17_3, D1_1, D1_2, D1_3, D14_1, D14_2, D14_3, D56_1, D56_2, D56_3, D98_1, D98_2, and D98_3) from breast muscle samples at six developmental stages yielding 10.1–20.6 million raw reads per library. After eliminating adaptors and low-quality reads, we obtained 5.0–16.3 million clean reads for these libraries (Table 1). These high-quality reads were mapped to chicken precursors in miRBase to identify known and novel miRNAs for further analysis. For all samples, the distribution of the small RNA sequence length was mainly concentrated at 22 nt, followed by 23 and 21 nt (Fig. 1).

**Table 1:** Statistics for the small RNA library sequences

Sample	Raw reads	Clean reads	18-26 nt reads	18-26 nt unique reads
E12_1	12,527,154	7,341,475	7,341,475	284,712
E12_2	15,074,636	10,210,965	10,210,965	301,091
E12_3	10,323,348	5,013,325	5,013,325	189,280
E17_1	15,822,038	11,317,278	11,317,278	223,975
E17_2	11,872,621	9,184,096	9,184,096	199,085
E17_3	15,438,410	14,271,465	14,271,465	235,002
D1_1	14,562,143	13,901,588	13,901,588	175,906
D1_2	11,206,695	9,370,394	93,703,94	127,933
D1_3	16,291,366	14,288,038	14,288,038	190,337
D14_1	16,927,396	14,074,485	14,074,485	1,080,281
D14_2	20,553,973	16,251,787	16,251,787	807,138
D14_3	16,639,718	13,318,345	13,318,345	476,165
D56_1	19,352,396	14,247,327	14,247,327	453,973
D56_2	15,171,865	11,909,307	11,909,307	433,093
D56_3	16,419,797	13,818,994	13,818,994	446,300
D98_1	10,635,824	7,959,557	7,959,557	314,128
D98_2	11,017,799	8,541,311	8,541,311	311,248
D98_3	10,144,518	8,296,123	8,296,123	229,398

### Differential expression of miRNAs

**Fig. 2:**
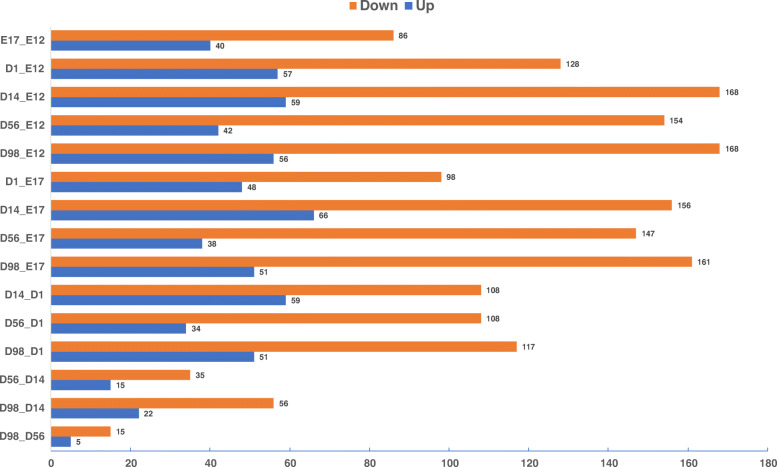
Numbers of upregulated and downregulated miRNAs in chicken breast muscle through pairwise comparisons

We identified 615 mature miRNAs corresponding to 401 precursor sequences based on the 18 small RNA libraries (Table S1), in which 337 miRNAs were differentially expressed during muscle development (Table S2). The number of downregulated miRNAs was higher than the number of upregulated miRNAs during development (Fig. 2). In pairwise comparisons, there were 126, 185, 227, 196 and 224 DE-miRNAs in E17, D1, D14, D56 and D98 compared with E12, respectively (Fig. 2). 126, 146, 167, 50 and 20 DE-miRNAs were found in E17 versus E12, D1 versus E17, D14 versus D1, D56 versus D14 and D98 versus D56, respectively (Fig. 2).

### STEM analysis of DE-miRNA expression profiles

**Fig. 3:**
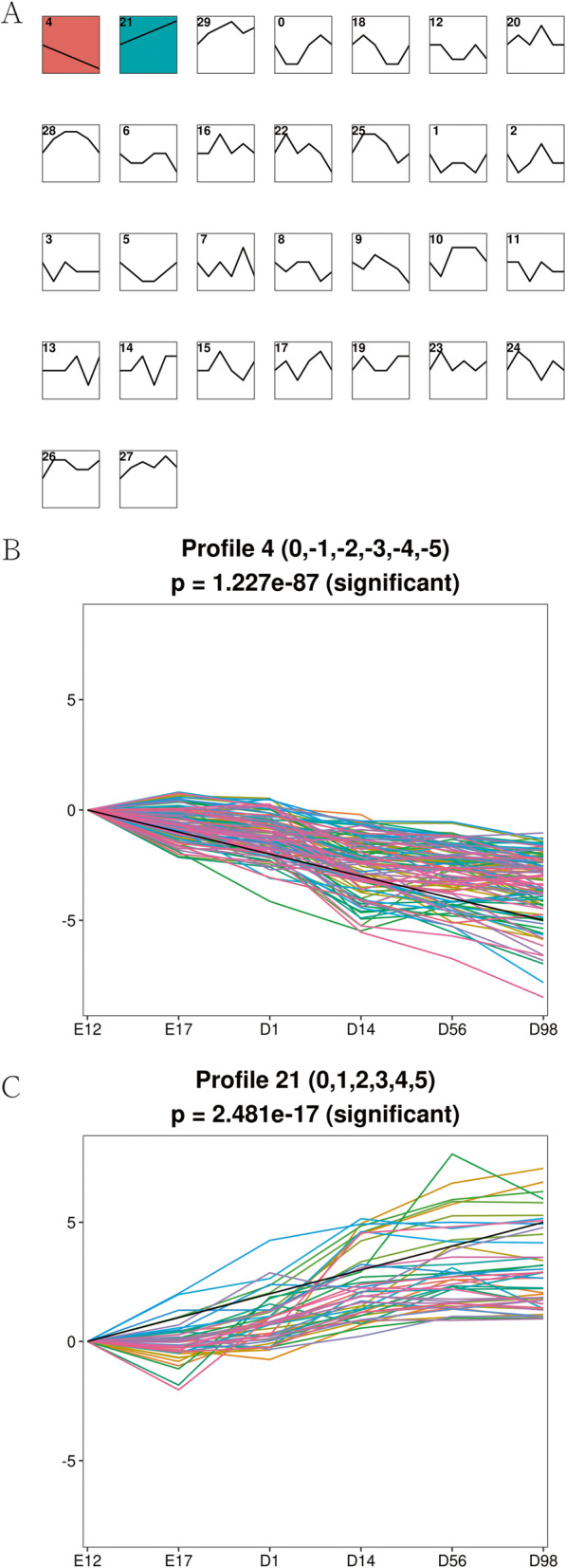
STEM analysis of DE-miRNA profiles. **a** Each box corresponds to a type expression profile and only colored profiles are significantly different. **b** Profile 4 with downregulated patterns. **c** Profile 21 with upregulated patterns

As our data were collected at different time-points, STEM was used to cluster and visualize possible changes in the profiles of 337 DE-miRNAs at six time points of breast muscle development. Within the 30 model profiles, two expression profiles containing 150 miRNAs were significant (*P*-value < 0.05, Fig. 3a). Of these, profile 4 with a downregulated pattern contained 106 DE-miRNAs (Fig. 3b, Table S3), while profile 21 with an upregulated pattern contained 44 DE-miRNAs (Fig. 3c, Table S4).

### Integrated analysis of DE-miRNAs and genes

In the previous section, profile 4 with 106 DE-miRNAs showed a downregulated pattern (Fig. 3b, Table S3), while profile 21 with 44 DE-miRNAs showed upregulated pattern (Fig. 3c, Table S4) by the STEM analysis. We explored the profiles of the differentially expressed protein-coding genes (DEGs) in breast muscle at E12, E17, D1, D14, D56 and D98 in a previous study, and identified 3233 downregulated and 380 upregulated DEGs (Table S5). It is a well-known fact that miRNA downregulate the expression of their target genes [17]. Therefore, the interactions of 106 downregulated DE-miRNAs and 380 upregulated DEGs or 44 upregulated DE-miRNAs and 3233 downregulated DEGs were predicted by miRBase (http://www.mirbase.org) and Targetscan software (http://www.targetscan.org) (free energy <− 10 kcal/mol and the pairing score > 50).

**Fig. 4:**
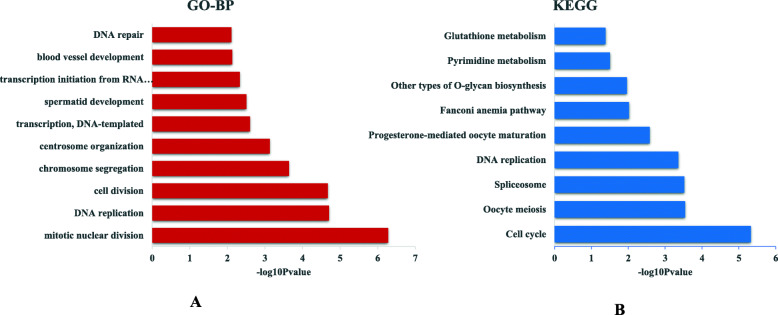
Functional annotation of miRNAs with upregulated patterns. **a** The significantly enriched biological process terms. **b** The significantly enriched pathways

For upregulated miRNA/downregulated protein-coding gene pairs, 4491 interactions were detected between 35 miRNAs and 1240 protein-coding genes (Table S6). GO analysis of the miRNA targets was performed to explore their functions. We found 70 GO terms that were significantly enriched (*P* < 0.05; Table S7), and most of these terms were associated with regulation of cell turnover. For example, the top five enriched biological process (BP) terms included mitotic nuclear division, DNA replication, cell division, chromosome segregation, and centrosome organization (Fig. 4a). KEGG analysis was significantly enriched in nine pathways (*P* < 0.05; Table S8); several of which were also related to cell turnover such as cell cycle, spliceosome, DNA replication, and pyrimidine metabolism (Fig. 4b).

**Fig. 5:**
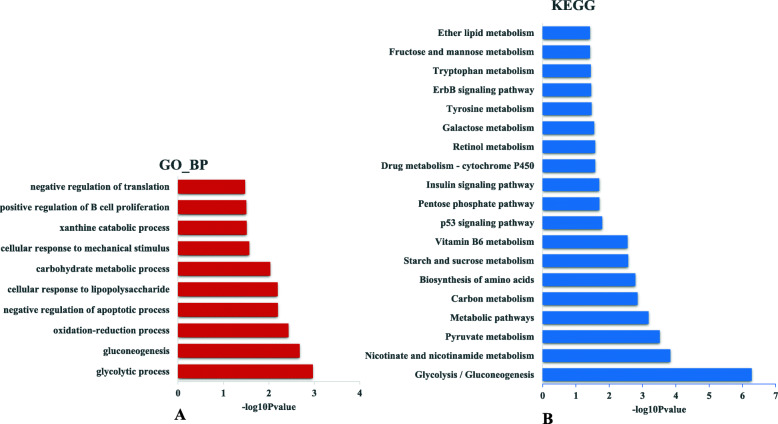
Functional annotation of miRNAs with downregulated patterns. **a** The significantly enriched biological process terms. **b** The significantly enriched pathways

For downregulated miRNA-upregulated gene pairs, 1873 interactions were detected between 91 miRNAs and 177 protein-coding genes (Table S9). Functional analysis of the miRNA targets showed that 18 GO terms were significantly enriched (*P* < 0.05) (Table S10), and some of the terms were related to metabolism, such as glycolytic process, gluconeogenesis, oxidation–reduction process, carbohydrate metabolic process, and xanthine catabolic process (Fig. 5a). In addition, 19 KEGG pathways were significantly enriched (*P* < 0.05; Table S11); several of which were also related to metabolism, including glycolysis/gluconeogenesis, pyruvate metabolism, carbon metabolism, biosynthesis of amino acids, pentose phosphate pathway, and insulin signaling pathway (Fig. 5b).

### Verification of the interaction between miRNA and target gene

**Fig. 6:**
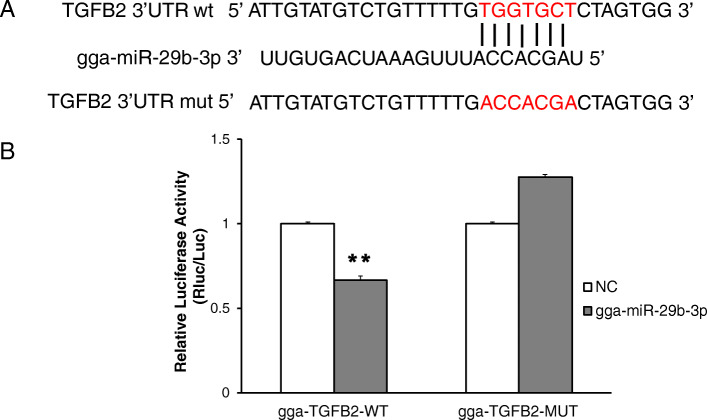
Identification of *TGFB2* as direct target of miR-29b-3p. **a** Schema of miR-29b-3p binding site in chicken *TGFB2* 3′-UTR sequence. **b** Target validation using a luciferase reporter assay

It has been reported that *TGFB2* plays an important role in regulating muscle development [18]. Our network analysis predicted that *TGFB2* is a target of four miRNAs: gga-miR-145-5p, gga-miR-29b-3p, gga-miR-2184a and gga-miR-6660 (Table S5). It has been demonstrated that miR-29b-3p is an important regulator of muscle development [19]. miR-29b-3p and *TGFB2* had opposite expression patterns during muscle development in the present study. Therefore, the target relationship between miR-29b-3p and *TGFB2* was validated using a luciferase reporter gene assay. As demonstrated in Fig. 6, miR-29b-3p significantly reduced the firefly luciferase activity of the wild type of the *TGFB2* reporter compared with negative control, suggesting that miR-29b-3p directly targets chicken *TGFB2* UTR.

### Validation of DE-miRNAs by qPCR

**Fig. 7:**
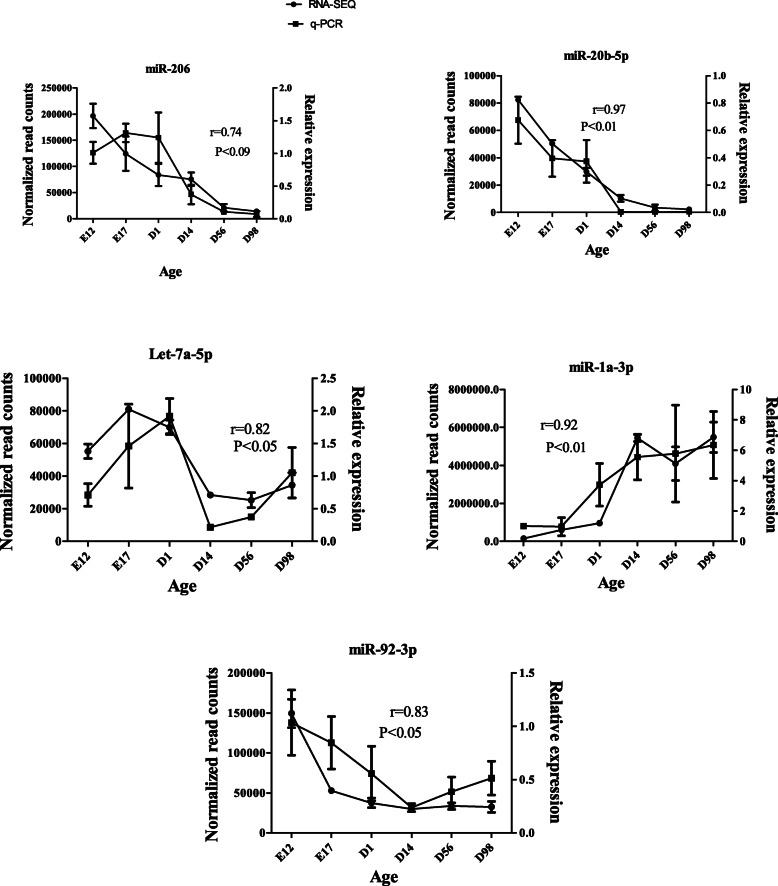
Validation of five DE-miRNAs by qPCR. The *r* value represents Pearson’s correlation coefficient between two methods

To validate the sequencing data, five DE-miRNAs (miR-1a-3p, miR-20b-5p, miR-206, miR-92–3p, and Let-7a-5p) were selected for qPCR analysis. Expression changes of qPCR data were significantly (*r* = 0.82–0.97, *P* < 0.05) correlated with sequencing data except for miR-206 (*r* = 0.74, *P* < 0.09) (Fig. 7), suggesting that our sequencing data were reliable.

## Discussion

Skeletal muscle development is a well-orchestrated process primarily controlled by many genes, transcription factors, ncRNAs and signaling pathways [4]. miRNAs as important post-transcriptional regulators play essential roles in fine tuning gene expression dynamics [5, 6]. However, there has been a lack of comprehensive studies about the dynamics of miRNAs across chicken muscle development. Only Jebessa et al. (2018) explored the miRNA expression profile during the chicken leg muscles development at E11, E16 and D1, and Li et al. (2019) analyzed miRNA and mRNA expression profiles during chicken breast muscle development at 6, 14, 22 and 30 weeks of age [15, 16]. We previously explored mRNA expression dynamics across chicken muscle developmental stages and found that there were distinct expression profiles in embryonic and post-hatching periods [14]. Therefore, to conduct a comprehensive study of miRNA expression dynamics and highlight key properties of miRNAs during chicken muscle development, we explored the expression patterns of miRNAs in chicken breast muscle from embryonic to post-hatching periods. We obtained 337 DE-miRNAs in pairwise comparisons between the libraries at the six developmental stages (Table S2). The regional differences in miRNA expression were greater during the early (e.g. E17 vs E12 and D1 vs E17) than late (e.g. D56 vs D14 and D98 vs D56) developmental stages and the greatest differences occurred when comparing D14 and D1. These results suggest that the time before and after hatching may be crucial for chicken muscle development.

Since our data were collected at different time points, we used STEM software, which is widely used to study dynamic biological processes [20], to investigate the dynamic miRNA changes during breast muscle development. Two profiles were found that better captured the expression patterns of DE-miRNAs (Fig. 3). Profile 4 with a downregulated pattern contained 106 DE-miRNAs (Fig. 3b), while profile 21 with an upregulated pattern contained 44 DE-miRNAs (Fig. 3c). There were more downregulated miRNAs, suggesting that the miRNAs are more active during the early developmental stages. Our previous study identified 3233 downregulated and 380 upregulated differentially expressed protein-coding genes in breast muscle at the same time point as in the present study (Table S5). It is a well-known fact that miRNAs mainly downregulate the expression of their target genes [17]. Therefore, we constructed the regulatory networks using the protein-coding genes and miRNAs with opposite expression patterns and performed GO and KEGG analysis of the miRNA targets to explore the function of candidate miRNAs.

**Fig. 8:**
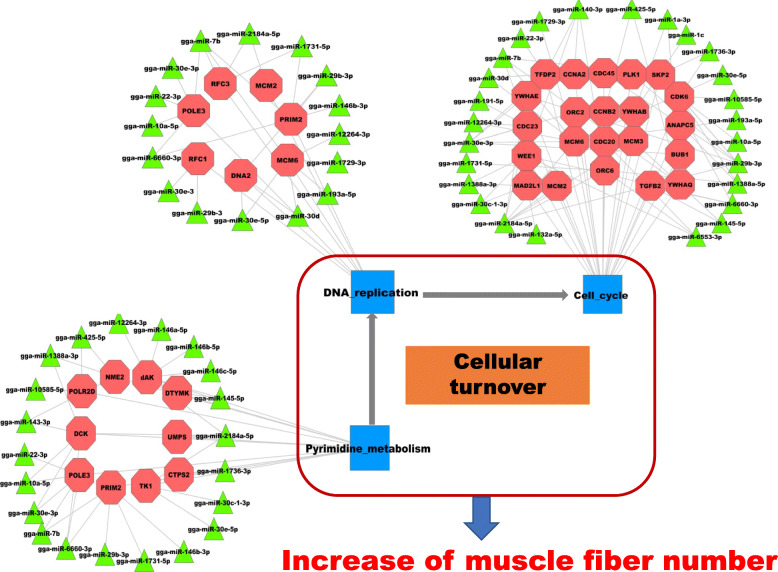
Predicted biomathematical miRNA–gene network among upregulated miRNAs and downregulated protein-coding genes. Green triangles represent upregulated miRNAs, whereas red octagons represent downregulated protein-coding genes

For the upregulated miRNA/downregulated protein-coding gene group, 35 upregulated miRNAs potentially targeted 1240 downregulated protein-coding genes (Table S6). Functional analysis showed that the miRNA targets were mainly involved in pyrimidine metabolism, DNA replication, and the cell cycle (Fig. 4). Pyrimidine metabolism is an important source of raw materials for DNA replication, while the cell cycle is accompanied by DNA replication, which are all related to cellular turnover. The growth of skeletal muscle mass depends on cellular turnover (differentiation and proliferation) and protein turnover (synthesis, degradation, and repair capacities) [10]. Cellular turnover plays a major role in embryonic muscle development [13]. Since miRNAs have been demonstrated to regulate gene expression negatively by translational repression and target mRNA degradation, the lower expression level of miRNAs that regulate genes of cellular turnover in embryonic periods suggests that cellular turnover plays a key role in embryonic muscle development. miRNA–target interactions that are involved in cellular turnover were integrated to construct possible regulatory networks, including 31 miRNAs (green triangle) and 35 targets (red octagon) (Fig. 8). Several target genes that were related to cellular turnover have been demonstrated to regulate muscle development, such as *CDC20*, *CNNA2*, *CNNB2*, *TGFB2*, *YWHAQ* and *YWHAE*. Cell division cycle gene *CDC20* regulates the proliferation of muscle precursor cells through directly targeting *Pax7* and *Pax3/7BP* [21]. Constitutive expression of *CCNA2* in transgenic mice yields robust postnatal cardiomyocyte mitosis and hyperplasia [22]. *CCNB2* also has a regulatory role in chicken breast muscle development [16]. The transforming growth factor-β superfamily encompasses a large group of growth and differentiation factors that play important roles in regulating embryonic development, and miR-599 can inhibit muscle cell proliferation by targeting *TGFB2* [18]. Our result demonstrated that miR-29b-3p might influence the muscle development through targeting *TGFB2* gene. *YWHA* has a role in vertebrate development and cell-cycle regulation [23]. The expression level of *YWHAQ* was significantly higher in porcine fetal muscle than adult muscle [24], and *YWHAE* was found to be involved in the longissimus dorsi muscle development of Hainan Black goats [25]. Several miRNAs interact with these genes, such as miR-1a-3p, miR-1c, miR-10a-5p, miR-22–3p, miR-29b-3p, miR-30e-3p, miR-30e-5p, miR-140-3p, miR-143-3p, miR-145-5p, miR-146a-5p, miR-146b-3p, miR-146b-5p, miR-146c-5p, miR-191-5p, and miR-193a-5p, and are implicated in muscle development and myogenesis regulation [3, 5, 26–29]. For example, the miR-1 family, the so-called muscle miRNAs, are abundant in muscle, and play key roles in skeletal muscle development [30]. miR-1 can reduce *CNND1* expression and repress myoblast proliferation [31]. miR-30 family miRNAs can modulate activity of muscle-specific miR-206 and protein synthesis by targeting *TNRC6A* [32]*.* miR-146b-3p acts in the proliferation, differentiation and apoptosis of myoblasts by directly suppressing the PI3K/AKT pathway and *MDFIC* in chickens [33]. All the above results show that the regulatory network consisting of these miRNAs and their targets might play important roles in muscle development through influencing cellular turnover. However, functional roles of some miRNAs in muscle development are unknown. Therefore, further experiments need to explore the mechanism of these miRNAs and their targets in regulation of muscle development.

**Fig. 9:**
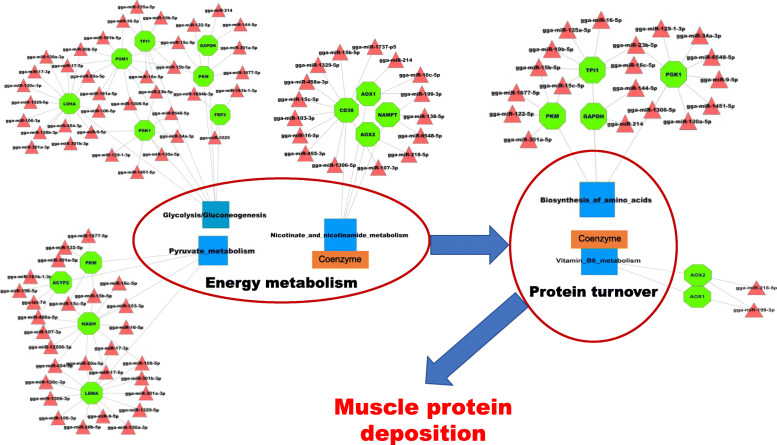
Predicted biomathematical miRNA–gene network among downregulated miRNAs and upregulated protein-coding genes. Red triangles represent downregulated miRNAs, whereas green octagons represent upregulated protein-coding genes

For the downregulated miRNA/upregulated protein-coding gene group, 1873 interactions were detected between 91 miRNAs and 177 protein-coding genes (Table S9). Functional analysis showed that the miRNA targets were mainly involved in protein turnover metabolism (Fig. 5). For example, glycolysis/gluconeogenesis and pyruvate metabolism can provide energy and materials for biosynthesis of amino acids, while the metabolites of nicotinate and nicotinamide metabolism are important coenzymes for energy metabolism, such as NAD^+^ and NADP^+^. The metabolites of vitamin B6 are also important coenzymes for biosynthesis of amino acids. The lower expression level of miRNAs that regulate genes of protein turnover in post-hatching periods suggests that protein turnover plays a key role in post-hatching muscle development, which is consistent with the previous study in which postnatal muscle development was mainly associated with accumulation of muscle-specific proteins [13]. Therefore, our results confirm that protein turnover plays an important role in postnatal muscle development at miRNA levels. miRNA–target interactions involved in protein turnover were integrated to construct possible regulatory networks, including 48 miRNAs (red triangle) and 13 targets (green octagon) (Fig. 9). Most target genes have been reported to have roles during muscle development. For example, as a glycolysis enzyme encoding gene, expression of *TPI1* was demonstrated to be positively correlated with the growth period in chicken and pig muscle [34–36]. Large white pigs store greater amounts of glycogen and induce higher expression of *PGM1*, *GAPDH*, and *LDHA* genes than Basque pigs do, which is one of the reasons why the former accumulate more lean meat than the latter [37]. *AOX1*, which encodes the glucose synthesis accelerating enzyme, has higher expression in broilers than in layers, and can contribute to myogenesis by influencing the level of H_2_O_2_ [38, 39]. Nicotinamide phosphoribosyl transferase (*Nampt*) has been identified as a rate-limiting NAD^+^ biosynthetic enzyme. *Nampt* can alter the expression of key myogenic transcription factors and may influence postnatal myogenesis [40]. Among the 48 miRNAs, more than half have been reported to have roles during muscle development, such as let-7d, miR-103-3p, miR-106-3p, miR-130b-3p, miR-16-5p, miR-16c-5p, miR-199-3p, miR-19b-5p, and miR-9-5p [41–48]. Although the effects of some miRNAs on muscle development are still unclear, such as miR-1306-5p, miR-1329-5p, miR-1451-5p, miR-1677-5p, and miR-1684b-3p, analysis of their targets demonstrated that they are involved in muscle development, suggesting that these miRNAs participate in regulating muscle development through regulating their target genes.

## Conclusion

We performed a comprehensive investigation of miRNA expression patterns in skeletal muscle in chickens across various developmental processes, from embryonic to adult stages. We identified 337 DE-miRNAs that were associated with chicken muscular development. Time series expression profile clustering analysis identified two significantly different expression profiles. miRNAs with an upregulated pattern were mainly involved in cellular turnover, while miRNAs with a downregulated pattern mainly played a regulatory role in protein turnover metabolism. These findings enhance our understanding of the regulatory mechanisms involved in muscle development at the miRNA expression level, and provide several candidates for future studies concerning miRNA–target function on regulation of chicken muscle development.

## Methods

### Ethical statement

All of the animal experiments were conducted in accordance with the Guidelines for Experimental Animals, established by the Ministry of Science and Technology (Beijing, China). Animal experiments were approved by the Science Research Department of the Shandong Academy of Agricultural Sciences (SAAS) (Ji’nan, China). Ethical approval for animal survival was given by the Animal Ethics Committee of SAAS (No. SAAS-2019-029).

### Animals

Shouguang chicken eggs were obtained from the Experimental Farm of the Poultry Institute (PI), Shandong Academy of Agricultural Sciences (SAAS, Ji’nan, China). All eggs were incubated with the normal procedure and chicks were reared in cages under continuous lighting using standard conditions of temperature, humidity and ventilation at the farm of the PI, SAAS. These chicks were allowed by the farm owners to be used in the present experiment. The same diet was fed to all chickens and a three-phase feeding system was used: starter ration (D1–D28) with 21.0% crude protein and 12.12 MJ/kg; the second phase (D28–D56) with 19.0% crude protein and 12.54 MJ/kg; and the last phase (> D56) with 16.0% crude protein and 12.96 MJ/kg. Feed and water were provided ad libitum during the experiment. Eighteen female chickens from the six developmental stages (E12, E17, D1, D14, D56 and D98) were used for sequencing. Three biological replicates for each stage. After incubating for 12 or 17 days, the eggs were broken with tweezers, the embryos were removed and electrically stunned and killed. Chickens after hatching were killed by stunning and exsanguination [49, 50]. All fresh breast muscle tissue samples were removed with scissors, frozen in liquid nitrogen, and stored at − 80 °C until RNA extraction. The sex of chicken embryos was identified by polymerase chain reaction (PCR) of the *CHD1* gene [51]. Chickens with two bands of 600 and 450 bp were born as female, and those with one band of 600 bp were born as male.

### Small RNA library construction and sequencing

Total RNA was extracted using TRIzol reagent (Invitrogen, Carlsbad, CA, USA) as described previously [52]. Each stage had three samples, and the total 18 samples (six groups × three samples/group = 18) used for further experiments. The total RNA quantity and purity were analyzed using Bioanalyer 2100 (Agilent, Santa Clara, CA, USA) with RNA integrity number > 7.0. Approximately 1 μg total RNA was used to prepare a small RNA library according to the protocol of Tru Seq Small RNA Sample Prep Kits (Illumina, San Diego, CA, USA). miRNA sequencing (HiSeq 2500 single end, 50 bp) was performed to identify miRNA species that are involved in the muscle development.

### Identification of differentially expressed (DE) miRNAs at different developmental stages

Raw reads were subjected to an in-house program, ACGT101-miR (LC Sciences, Houston, TX, USA) to remove adapter dimers, junk, low complexity sequences, common RNA families (rRNA, tRNA, small nuclear RNA and small nucleolar RNA) and repeats. Subsequently, unique sequences of 18–26 nt were mapped to chicken precursors in miRBase 22.0 (http://www.mirbase.org/) [53] by BLAST search to identify known miRNAs and novel 3p- and 5p-derived miRNAs. Length variation at 3′ and 5′ ends and one mismatch inside of the sequence were allowed in alignment. The unique sequences mapping to chicken mature miRNA in hairpin arms were identified as known miRNAs. The unique sequences mapping to the other arm of the known chicken precursor hairpin opposite the annotated mature miRNA-containing arm were considered to be novel 5p- or 3p-derived miRNA candidates. The remaining sequences were mapped to other selected species precursors (with the exclusion of chicken) in miRBase 22.0 (http://www.mirbase.org/) [53] by BLAST search, and the mapped pre-miRNAs were further BLASTed against the chicken genome (Gallus_gallus 5.0) to determine their genomic locations. miRNAs obtained from the above two analyses were defined as known miRNAs. The unmapped sequences were BLASTed against the chicken genome (Gallus_gallus 5.0), and the hairpin RNA structures containing sequences were predicated from the flanking 80 nt sequences using RNAfold software (http://rna.tbi.univie.ac.at/cgi-bin/RNAfold.cgi). Modified reads per million were used to quantify the normalized reads. The formula was: Normalized Expression = Actual miRNA count/Total count of clean reads. Differentially expressed miRNAs were identified through pairwise comparisons between every two stages (E17 vs E12, D1 vs E12, D14 vs E12, D56 vs E12, D98 vs E12, D1 vs E17, D14 vs E17, D56 vs E17, D98 vs E17, D14 vs D1, D56 vs D1, D98 vs D1, D56 vs D14, D98 vs D14, and D98 vs D56).. The DE-miRNAs based on normalized counts were analyzed using Student’s *t* tests according to the experimental design, and the significance threshold was set as *P* < 0.05.

### Time-series expression profile clustering

The non-parametric clustering algorithm of Short Time-Series Expression Miner (STEM version 1.3.11) [20] was used to cluster and visualize possible profiles and changes in expression over time in DE-miRNAs. The maximum unit change in model profiles between time points was adjusted to 1 and the maximum number of model profiles to 30. STEM was run using the log normalized data option, with all other settings set to the defaults. The statistical significance (*P* < 0.05) of the number of DE-miRNAs to each profile versus the expected number was computed using the algorithm from a previous study [20].

### Integrated analysis of DE-miRNAs and genes

We explored the profiles of differentially expressed genes (DEGs) in Shouguang chicken breast muscle as described previously [14], and identified 9447 DEGs. It is a well-known fact that miRNA downregulate the expression of their target genes. Therefore, we firstly used STEM to cluster and visualize possible profiles and changes in expression over time in DE-miRNAs and DEGs, and then selected the DEGs and DE-miRNAs with opposite expression patterns to predict- their interactions by miRBase (http://www.mirbase.org) and Targetscan software (http://www.targetscan.org) (free energy <− 10 kcal/mol and the pairing score > 50). miRNA–gene interactions were integrated to construct a possible regulatory network using Cytoscape (http://cytoscape.org/). The Gene Ontology (GO) analysis of DE-miRNA targets was performed using the Database for Annotation, Visualization and Integrated Discovery (DAVD; http://david.abcc.ncifcrf.gov/) [54]. The Kyoto Encyclopedia of Genes and Genomes (KEGG) pathway analysis was used KOBAS version 3.0 [55]. GO terms and KEGG pathways with *P* < 0.05 were considered significantly enriched.

### Cell culture

293 T (human embryonic kidney) cells obtained from ATCC (Cell Systems & cGMP Biorepository, Gaithersburg, MD, USA) and were cultured in DMEM (Gibco, Gaithersburg, MD, USA) supplemented with 10% fetal bovine serum (Hyclone, Logan, UT, USA), 1% penicillin/streptomycin (Invitrogen, Carlsbad, CA, USA). All cells were cultured at 37 °C in a 5% CO2 humidified atmosphere.

### Dual-luciferase reporter assay

The pmiR-RB-Report™ (RiboBio, Guangzhou, China) including double luciferase reporter genes was used to test and validate the target sites for gga-miR-29b-3p. The 3′ UTR of *TGFB2* containing gga-miR-29b-3p binding sites were amplified from chicken genomic DNA. All PCR products were cloned into the pmiR- RB-Report Vector using Xhol and NotI restriction enzymes. Wild-type or mutant TGFB2–3′ UTR dual-luciferase reporter (200 ng) and miR-29b mimic or NC duplexes (50 nM) were co-transfected into 293 T cells using the Lipofectamine 3000 reagent (Invitrogen, Carlsbad, CA, USA) in 48-well plates. Firefly and Renilla luciferase activities were measured at 48 h post transfection using a Dual-GLO Luciferase Assay System Kit (Promega, Madison, USA), following the manufacturer’s instructions. Luminescence was measured using a Fluorescence/Multi-Detection Microplate Reader (BioTek, Vermont, USA) and firefly luciferase activities were normalized to Renilla luminescence in each well.

### Quantitative real-time PCR

To validate and characterize the DE-miRNAs identified via high-throughput sequencing, quantitative PCR (qPCR) analyses were performed in an ABI 7500 Detection System (Applied Biosystems, Foster, CA, USA). The expression level of miRNA (miR-206, miR-20b-3p, miR-1a-3p, miR-92–3p, let-7a-5p) was determined using Bulge-Loop miRNAs qPCR Primer Set (RiboBio Biotechnology, Guangzhou, China). cDNA was synthesized with a miRNA-specific stem-loop primer, while qPCR was performed with the specific primers using KAPA SYBR® FAST qPCR Kits (Wilmington, MA, USA). The U6 small nucleolar RNA gene was used as an internal control. The relative miRNA expression level was calculated using the 2^-ΔΔCt^ method [56], and data were expressed as means ± SD. The pearson correlation between relative expression from qPCR and normalized read counts from RNA-seq was analyzed using SPSS 16.0.

## Supplementary Information

**Additional file 1: Table S1**. Expression of all identified miRNAs

**Additional file 2: Table S2**. DE-miRNAs

**Additional file 3: Table S3**. miRNAs of profile 4

**Additional file 4: Table S4**. miRNAs of profile 21

**Additional file 5: Table S5**. Downregulated and upregulated protein coding genes

**Additional file 6: Table S6**. Interaction between upregulated miRNAs and downregulated genes

**Additional file 7: Table S7**. GO terms of upregulated miRNA targets

**Additional file 8: Table S8**. KEGG pathways of upregulated miRNA targets

**Additional file 9: Table S9**. Interaction of downregulated miRNAs and upregulated genes

**Additional file 10: Table S10**. GO terms of downregulated miRNA targets

**Additional file 11: Table S11**. KEGG pathways of downregulated miRNA targets

## Data Availability

The raw sequence data reported in this paper have been deposited in the Genome Sequence Archive in BIG Data Center, Beijing Institute of Genomics (BIG), Chinese Academy of Sciences and is publicly accessible at http://bigd.big.ac.cn/gsa (accession no CRA002587).

## Abbreviations

AOX1: Aldehyde oxidase 1
CCNA2: cyclin A2
CCNB2: Cyclin B2
CCND1: Cyclin D1
CDC20: Cell division cycle 20
GAPDH: glyceraldehyde-3-phosphate dehydrogenase
LDHA: lactate dehydrogenase A
MDFIC: MyoD family inhibitor domain containing
Nampt: Nicotinamide phosphoribosyltransferase
Pax7: Paired box 7
Pax3/7BP: PAX3 and PAX7 binding protein 1
PGM1: Phosphoglucomutase 1
TGFB2: Transforming growth factor beta 2
TNRC6A: Trinucleotide repeat containing adaptor 6A
TPI1: Triosephosphate isomerase 1
YWHA: Tyrosine 3-Monooxygenase/Tryptophan 5-Monooxygenase Activation Protein
YWHAE: Tyrosine 3-monooxygenase/tryptophan 5-monooxygenase activation protein epsilon
YWHAQ: Tyrosine 3-monooxygenase/tryptophan 5-monooxygenase activation protein theta
